# Proteomics and melanoma: a current perspective

**Published:** 2016-06-20

**Authors:** Bradley D Shields, Alan J Tackett, Sara C Shalin

**Affiliations:** 1 Department of Biochemistry and Molecular Biology, University of Arkansas for Medical Sciences, Little Rock, AR, USA; 2 Departments of Pathology and Dermatology, University of Arkansas for Medical Sciences, Little Rock, AR, USA

## Abstract

Proteomics is the study of the protein complement of the genome, and this powerful technique complements genomic studies. Proteomic experiments result in the generation of large volumes of data requiring complicated analysis algorithms and subsequent confirmatory studies. Until recently, technological limitations of experimental protocols precluded the use of formalin-fixed tissues for these types of studies. Recent advances have allowed the use of valuable archived patient tissue samples in proteomic research, resulting in an opportunity to perform cutting edge translational research. The field of melanoma research stands to benefit greatly from collaboration between dermatopathologists and proteomic scientists. This article seeks to: 1) describe proteomics for dermatologists and pathologists, including the tools used in proteomic research, and 2) convey a historical account of proteomic studies within the field of melanoma followed by a discussion on how recent advances are informing current studies.

## Introduction

A new science often first grows in leaps and bounds but eventually the rate of knowledge expansion levels off until new technological advancements or novel discoveries trigger a rapid growth episode. The field of proteomics is currently in the “leaps and bounds” stage of its development. Proteomics is the study of the protein complement of the genome, and it is finding wide application in translational medicine. The field of proteomics seeks first to identify and quantify proteins in a given sample, but ultimately to also determine the proteins’ modifications, interactions with other proteins, and functions. The ability to characterize the proteome of normal and diseased cells is increasingly being viewed as an asset in the development of personalized medicine, due to the fact that studies of genetics or gene expression are several levels of control removed from the proteins they code for—the true effectors of cellular behavior. Here, we aim to discuss how advances in the field of proteomics have impacted and are impacting melanoma research. We will demonstrate the development and course of the field, with particular emphasis on how proteomic discoveries can play a role in the development of predictive biomarkers and targeted drug development.

## Proteomics: the basics

**Figure 1:**
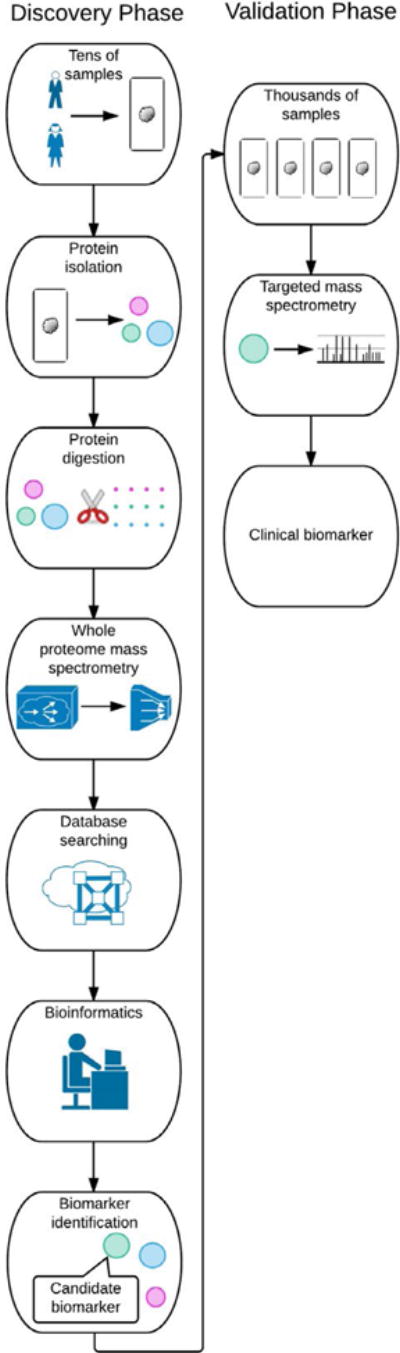
Schematic diagram of discovery phase and validation phase proteomics.

Proteomic applications in biomedical research can largely be categorized into discovery and targeted validation phases. Discovery phase proteomics refers to the large-scale identification and quantification of proteins or protein posttranslational modifications from a complex biological sample such as cells in culture, tissues or plasma. High resolution and rapid-sampling mass spectrometers that are operating in a data-dependent mode (*i.e.*, collecting data in real time) are the foundation of discovery phase proteomics. Discovery phase proteomics generally utilizes 10’s of samples and generates 1000’s of candidate proteins for follow-up studies. Targeted validation proteomics typically follows the discovery phase. For targeted validation proteomics, specialized mass spectrometers are used to specifically quantify the levels of a small set of proteins in a large number of samples. Targeted validation proteomics generally utilizes 1000’s of samples to measure 10’s of target proteins. The specific types of mass spectrometers are different for discovery and targeted validation proteomics – accordingly, many proteomics core facilities are specialized in one or the other proteomic sub-specialty (Figure 1).

A variety of workflows exist for proteomic investigations, but the essential steps involve isolating a protein sample (cell lysate, serum, etc.), denaturing and resolving the proteins, and then enzymatically digesting the proteins into peptides (often with trypsin). Next, peptides are further separated via liquid chromatography and analyzed by mass spectrometry. A mass spectrometer measures mass to charge ratios (m/z) and consists of three principal components: the ion source, mass analyzer, and mass detector. Proteins are digested into peptides to make them more amenable to ionization, which is driven by methods such as MALDI (matrix-assisted laser desorption/ionization) or ESI (electrospray ionization), in which the sample is atomized by spraying it out of a highly charged fine-tipped needle). Next, the mass analyzer separates the ions suspended in the vacuum. Common mass analyzers include TOFs (time of flight), ion traps, and quadrupoles. Finally, the mass detector produces a signal when the ions selected to pass through the instrument strike the detector’s surface. State of the art mass spectrometers employ advanced liquid chromatography systems coupled to electrospray ionizers and multiple mass analyzers in tandem to provide high resolution. It is fair to draw an analogy between the dramatic advancement in processing power of computers over the last few decades and the capabilities of advanced cutting-edge mass spectrometers compared to their predecessors. The resulting spectral data, corresponding to protein signals, are interpreted by software programs which assign protein identifications to peptide fragments, and this is how the protein content of a sample is determined. Advancements in proteomics over the past five years cover the entire spectrum of the discipline and include improved methods of sample preparation, drastically more powerful instruments and computer hardware, more powerful software programs to analyze data, and most importantly, heightened participation by the clinical community in supplying patient samples for investigation. Proteomic studies, then, are able to determine the relative abundance of proteins in various samples, the modifications of these proteins or their levels with various treatments, or the comparative fractions of proteins between two related samples.

Validation of mass spectrometry data from discovery phase proteomics is a critical component of proteomic investigations. Once a proteomic study has identified candidate proteins of interest in the samples being analyzed, confirmatory testing becomes necessary and targeted validation proteomic studies and systems biology generally provide methodologies necessary to validate and reveal mechanisms underlying the results of cell-line discovery studies. Once target proteins are identified, available validation methods include RT-PCR to analyze mRNA expression of the protein, western blots on cell lysates to determine quantification of protein expression, or protein immunohistochemistry to localize and quantify protein expression. Mechanisms underlying a difference in protein expression may be further explored using “knock out” or “knock down” techniques or treatment with a targeted inhibitor drug performed first in cell lines, and ultimately in rodent models.

## Melanoma: a disease well-suited for proteomic studies

Physicians require better and more defined protein markers that may diagnose, provide prognostic information, and assist in the selection of therapeutic strategies for melanoma. Despite a rise in the public’s awareness of the damaging effects of the sun, the incidence of melanoma continues to increase worldwide [1]. Melanoma is still rare, representing just 5% of all skin cancer cases, but it is deadly, accounting for 75% of deaths from skin cancer [2]. Melanoma arises from melanocytes, the melanin pigment–producing cells of the epidermis, whose normal function is to secrete melanin to protect the skin from UV-radiation damage [3]. Melanoma is often detected clinically, distinguished from benign nevi by visual characteristics including size, asymmetry in shape, color, and border, and documented clinical growth and can easily be biopsied and removed by surgical excision. Upon microscopic examination, melanomas have traditionally been divided into four predominant histopathologic subtypes (superficial spreading melanoma, lentigo maligna melanoma, nodular melanoma, and acral lentiginous melanoma) based upon their histopathologic characteristics, despite the historic inability of histologic patterns to inform prognosis. Indeed, the most important prognostic parameter at the time of biopsy is the depth of tumor invasion into the dermis (Breslow’s depth). The presence of ulceration and the presence of mitotic figures in thin melanomas also are significant prognostic factors that correlate with overall survival and impact the staging of melanoma based on the current 7^th^ edition of AJCC staging manual [4]. Other histopathologic features that are known to impact survival but to a lesser degree include the anatomic level of the skin (Clark level), presence of tumor-infiltrating lymphocytes, and regression. Perhaps intuitively, regional lymph node involvement and the presence of distant metastasis correlate strongly with poor outcome and also impact clinical and pathologic staging of the disease [3].

Recently, investigations into the molecular mechanisms driving melanoma have provided insight into the truly diverse disease encompassed by the term “melanoma”, and there is a growing recognition that the histopathologic subtypes may in fact correspond to certain molecular aberrations that drive the tumor formation [5,6]. Pathogenic genetic mutations in melanoma continue to be characterized, and the order in which tumors acquire mutations provides insight into the progression of *in situ* melanoma becoming invasive and ultimately widely metastatic disease [7]. While some of these mutations, such as the BRAF V600E mutation, provide druggable targets for metastatic disease, genetic mutations alone fail to capture the complexity and dynamic properties of this disease. In the face of a relative paucity of diagnostic, prognostic, and therapeutic markers, proteomics offers a way in which to better evaluate this complex disease. To date, an array of proteomic investigations have been performed on cultured melanoma cell lines, melanocytes, and patient serum and tissue samples. A brief look into investigations of the last decade sets the context for studies in the past five years.

Proteomic studies in cell lines have provided valuable insight into proteins involved in melanoma development, progression, and responsiveness to therapeutic agents. In 2003, Bernard and colleagues examined cell lines of cultured melanocytes, primary melanoma, and metastatic melanoma for differences in protein expression that could be related to tumorigenesis, and found increased expression of nucleophosmin/B23 and hepatoma-derived growth factor in the melanoma cell lines [8]. Carta *et al.* in 2005 compared primary and metastatic melanoma cell lines with a goal of finding differences in protein expression that correlated with disease progression. Their results revealed dysregulation of a number of proteins within cellular stress pathways, including upregulation of several heat shock proteins in metastatic melanoma cell lines, suggesting that these proteins play a role in melanoma progression [9]. In a study aimed at recapitulating the *in vivo* microenvironment of tumors, Hood et al. seeded skin organ cultures with primary and metastatic melanoma cell lines. After growing for two weeks, melanoma cells were harvested with laser microdissection and analyzed by mass spectrometry. They found cell-matrix and cell-adhesion proteins were upregulated in melanomas when compared to normal melanocytes, suggesting ample crosstalk between melanoma cells and the tumor microenvironment [10]. Instead of trying to identify protein markers of melanoma development or progression, other studies have compared protein expression profiles of melanoma cell lines responsive versus cell lines non-responsive to various chemotherapies. Using this methodology, Sinha and colleagues identified 25 proteins of interest, which included chaperone proteins from the heat shock protein family [11]. These studies and others have generated a list of candidate proteins implicated in melanoma biology and mechanistic studies to support their importance. However, it should be noted that investigations of melanoma cell lines, while readily available and relatively easy to initiate, have several shortfalls as noted in a prior review by Sabel *et al.* [12]. Creation of a melanoma cell line for use in the laboratory is immediately biased to patients who have a harvestable (i.e. sufficiently large) tumor, and cell line generation sub-selects tumors that grow well in vitro. Furthermore, it is known that protein expression changes upon cell culture *in vitro* [12].

In lieu of an adequate source of tumor tissue for investigation, serum studies emerged in the mid-2000s that sought to work around challenges with cell lines. The entire volume of a human’s blood is pumped around the body roughly once a minute. Blood carries not only erythrocytes and plasma proteins, but also multiple proteins from other tissues and tumors as well. Mian *et al.* compared serum samples from patients with either stage I or stage IV melanoma, and successfully discriminated samples using protein chip technology [13]. Takikawa *et al.* compared the serum proteome between healthy volunteers and melanoma patients. Nine proteins were detectable in the plasma from the melanoma patients that were not found in normal patients’ plasma [14]. Platelet basic protein precursor (PPBP) was identified as a marker whose expression level corresponded with prognosis. Acquisition of serum samples comes with relatively few challenges, as blood draws are routine. However, performing proteomic analysis on serum samples has innate technical challenges. Roughly 97% of the proteins found in the plasma belong to just seven groups of highly abundant proteins including albumin, immunoglobulins, fibrinogen, alpha-1 antitrypsin, alpha 2 macroglobulin, transferrin, and lipoproteins. The low abundance of serum tumor proteins makes finding and quantifying them especially difficult. This challenge is compounded by the variable levels and transient nature of tumor proteins in serum. Greco *et al.* sampled serum from 50 patients undergoing biopsy for probable melanoma. Patients who were found to have no melanoma and no other malignancies were used as controls. Transthyretin and angiotensinogen were increased and vitamin D binding protein was decreased in patients who were diagnosed with melanoma [15]. However, one month after surgical removal of the melanoma, these proteins were no longer elevated; thus their transience limits their use as biomarkers. The moving target presented by tumor proteins in serum has limited potential translational approaches and strategies for clinical application.

While cell lines present incomplete biological fidelity and serum samples are hampered by complexity and transitory changes in the proteome, archival patient tissue samples may provide the needed study environment. Real patient tissues in the form of biopsies or excisions give an accurate snapshot of the proteome at a discrete point in the disease process. These samples are archived- often indefinitely-as formalin-fixed and paraffin-embedded (FFPE) specimens and are linked to clinical data that includes patient demographics, disease course, and outcome. As recent as 2011, a review on proteomics and melanoma noted that despite the ample supply of melanoma FFPE samples, “Unfortunately, FFPE tissues are typically refractory to proteomic investigations using today’s methodologies, largely due to the high level of covalently linked proteins arising from formalin fixation [12].”

However, about the same time, studies were published describing protocols which successfully extracted proteins from FFPE tissues for subsequent proteomic analysis. There is little doubt mass spectrometric analysis of FFPE tissues samples is still hampered by modifications caused by formalin fixation. However, work is ongoing to mine FFPE datasets using software programs that can search and correct data for the presence of specific modifications, providing for lines of inquiry into maximizing the potential of archival samples. Covalent linkage of proteins from formalin fixation may prove refractory to sequence analysis and their unpredictability may prove to be an innate characteristic of FFPE tissue proteomics. Nevertheless, insights into melanoma pathogenesis have already been gained from archival tissue proteomic studies.

In 2010 and 2011 respectively, Rezaul and Byrum both published proof-of-principle techniques on extraction of proteins from FFPE melanoma for subsequent mass spectrometry [16,17]. The technique includes laser and needle micro-dissection of tumor, followed by deparaffinization and reversal of formalin cross-linking via a series of washes and sonications. Next, proteins from each sample are resolved via SDS-PAGE and Coomassie-stained before in- gel digestion and mass spectrometric analysis. In 2013, Byrum *et al.* performed the most comprehensive quantitative proteomic study to date using FFPE human melanoma tissues [18]. Using 61 patient samples, the authors identified 171 of 1528 examined proteins that varied in abundance among benign nevi, primary melanoma, and metastatic melanoma. Seventy-three percent of these identified proteins were found to have been validated by protein immunohistochemistry of melanoma tissues as documented in the Human Protein Atlas database [18].

**Figure 2:**
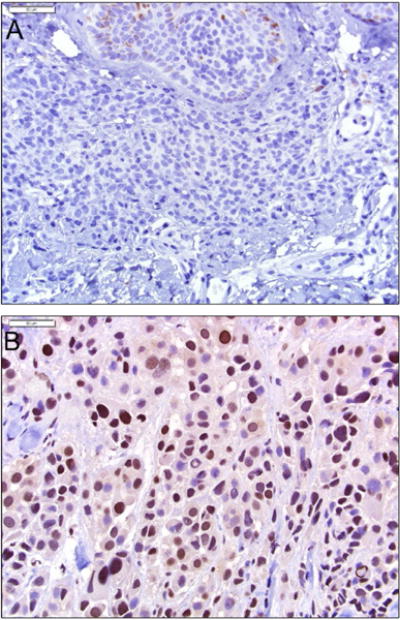
EZH2 expression in melanocytic neoplasms. EZH2 expression is absent in nevus cells (A), and upregulated in metastatic melanoma (B). Both images, 400×

Expanding on these data, in 2015, Sengupta and colleagues performed quantitative mass spectrometric analysis of histone posttranslational modifications using both melanoma cell lines and FFPE archival patient samples. They found increased histone H3 lysine 27 trimethylation (H3K27me3) was accompanied by over-expression of EZH2 (a histone methyltransferase) in both metastatic cell lines as well as in metastatic tumor FFPE tissues (Figure 2) [19]. Aberrant histone posttranslational modifications (PTMs) are considered crucial in the development and progression of human cancers and this was the first detailed study establishing epigenetic reprogramming of H3K27me3 as a major driver in melanoma progression. Examining histone PTMs with mass spectrometry requires specialized workflows that include delicate extractions and targeted chemical modifications to preserve the covalent modifications to the proteins. Despite the complexity of PTM analysis, accumulating evidence calls for more proteomic studies of epigenetics in melanoma. For instance, Lian and colleagues have documented the loss of an epigenetic mark 5-hydroxymethylcytosine (5-hmC) in melanoma progression via immunohistochemical staining, tissue microarrays, and genome-mapping [20], a finding subsequently confirmed by another group [21]. Hypermethylation of the promoter regions (leading to transcriptional silencing) of genes such as PTEN and others have also been implicated in melanomagenesis [22,23], and large scale studies using next generation sequencing techniques have found a high frequency of somatic mutations in genes encoding proteins that regulate epigenetic modifications [24]. Unbiased proteomic discovery studies targeting epigenetic marks may help uncover additional epigenetic modifications and epigenetic modifiers present in melanoma which could ultimately serve as therapeutic targets and complement the ongoing investigation of the genetic landscape of melanoma.

A 2011 review by Sabel *et al.* levels fair criticisms against some melanoma “biomarker discovery” studies. One weighty critique is a lack of reproducibility across studies. This may reflect the previously mentioned concept that melanoma is actually a diverse disease, as reflected by the many different histologic patterns recognized under the microscope. Another strong criticism directed against tissue proteomics by the 2011 review is that proteomic studies that evaluate protein expression of metastatic tumors essentially focus on a population with a uniform poor prognosis in which biomarkers identified would be of limited utility [12]. However this is not still the case; the last five years have seen substantial progress in treatment efficacy. Discovery of mutations in the mitogen activated protein (MAP) kinase signal transduction pathway in about 50% of melanomas [25], led to the development of BRAF and MEK inhibitors, the first being vermurafenib, which became FDA approved in 2011 [26]. Responses to BRAF and MEK inhibitor therapy are initially profound but temporary, as virtually all patients suffer from emergence and proliferation of resistant tumor cells [27].

Development of immunotherapy, in the form of immune-modulating antibodies, has shown limited, but dramatic success in treating patients with metastatic melanoma. These antibodies, developed against CTLA-4 (ipilimumab) and PD-1 (pembrolizumab and nivolumab), release brakes on the immune system that normally serves to maintain homeostasis and prevent autoimmunity. Response rates for monotherapies with these drugs range from 10–30%, but when patients do respond, it is often in a durable and lasting way, in contrast to patients treated with BRAF and MEK inhibitors. Response rates for combination therapies are somewhat higher; ipilimumab and nivolumab combination was associated with a response rate of 57.6% [28]. Even with combination therapies, however, approximately 40% of patients do not respond to immune checkpoint inhibitor therapy, so continued investigations of therapeutic modalities is needed. Moreover, in addition to relatively low response rates, immunotherapies are costly ($150,000 USD for a one-year regimen of monotherapy) and significant autoimmune side effects have been reported. The likelihood of response to immune checkpoint inhibitor therapy is a new area for investigation in which fundamental clinical care questions are unanswered (and biologic causes unknown). It remains to be determined why some people respond to immune checkpoint inhibitor therapy while others do not, and it is yet unknown if there are molecular signatures that can predict responsiveness to immunomodulatory agents. These unanswered questions represent a ripe area for investigation by proteomic research, and the answers will become increasingly important in the current era of responsibly allocating limited medical resources. In order for such investigations to be successful the challenge of ensuring reproducibility of data is paramount. A few key biomarkers may exist relating to pathogenesis or that indicate therapeutic response that are widespread across large subpopulations of melanomas, but the complexity of the disease predicts that many of the biomarkers discovered by such studies apply only to a subset of melanomas.

In summary, proteomic studies have already begun to bring insight into melanomagenesis and disease progression, complementing and enriching genetic studies and conventional systems-based investigational approaches. Large scale proteomic research studies are also amenable to broader applications with regard to biomarker discovery and validation. Molecular biologists and pathologists working in this area now face the challenge of piecing together seemingly disparate results across studies in order to create a clearer picture of this complex disease. Robust interactions and collaborations between basic scientists, clinicans, and translational scientists will ensure that the intricacies of melanoma pathogenesis are respected and addressed comprehensively. Proteomics for melanoma biomarker discovery still faces immense challenges, but with new avenues for study and ongoing rapid advancement, it is fair to reason that the near future holds more discoveries.
